# Hepatoma-Derived Growth Factor and DDX5 Promote Carcinogenesis and Progression of Endometrial Cancer by Activating β-Catenin

**DOI:** 10.3389/fonc.2019.00211

**Published:** 2019-04-11

**Authors:** Chunhua Liu, Lijing Wang, Qingping Jiang, Junyi Zhang, Litong Zhu, Li Lin, Huiping Jiang, Dan Lin, Yanyi Xiao, Weiyi Fang, Suiqun Guo

**Affiliations:** ^1^Department of Obstetrics and Gynecology, The Third Affiliated Hospital, Southern Medical University, Guangzhou, China; ^2^Department of Pathology, Third Affiliated Hospital of Guangzhou Medical University, Guangzhou, China; ^3^Integrated Hospital of Traditional Chinese Medicine, Southern Medical University, Guangzhou, China

**Keywords:** EC, HDGF, DDX5, β-catenin, PI3K/AKT

## Abstract

**Background:** Our previous work determined the correlation between high nuclear expression of hepatoma-derived growth factor (HDGF) and clinicopathological data of endometrial cancer (EC); however, the modulatory mechanisms and biological role of HDGF in EC have not been reported.

**Methods:** Lentiviral particles carrying human HDGF short hairpin RNA (shHDGF-1, -2, and -3) vector and plasmids for HDGF, DDX5, and β-catenin expression were, respectively introduced into EC cells to evaluate the effects and molecular mechanisms underlying EC cell proliferation, migration, invasion, and metastasis. Quantitative real time reverse transcription polymerase chain reaction (qRT-PCR) and western blotting were used to determine HDGF and DDX5 expression. Co-immunoprecipitation (co-IP), mass spectrometry, and an immunofluorescence co-localization study were conducted to explore the relationship between HDGF, DDX5, and β-catenin. Immunohistochemistry was used to analyze the clinical associations between HDGF and DDX5 in EC.

**Results:** Knocking down HDGF expression significantly decreased EC cellular proliferation, migration, invasion *in vitro*, as well as tumorigenesis and metastasis *in vivo*. Conversely, HDGF overexpression reversed these effects. Stable knockdown-based HDGF suppression activated the PI3K/AKT signaling pathway, along with downstream β-catenin-mediated cell cycle and epithelial-mesenchymal transition signaling. Furthermore, co-IP combined with mass spectrometry and an immunofluorescence co-localization study indicated that HDGF interacts with DDX5, whereas β-catenin was associated with DDX5 but not HDGF. Overexpression of DDX5 reversed the suppression of shHDGF. Immunohistochemistry analysis showed that high expression of DDX5 constituted an unfavorable factor with respect to the clinicopathological characteristics of EC tissues and that HDGF and DDX5 high expression (HDGF+/DDX5+) led to a worse prognosis for patients with EC (*P* < 0.001). In addition, we found that the expression of HDGF and DDX5 was positively correlated in EC tissues (*r* = 0.475, *P* < 0.001).

**Conclusion:** Our results provide novel evidence that HDGF interacts with DDX5 and promotes the progression of EC through the induction of β-catenin.

## Introduction

Endometrial cancer (EC) comprises the most common malignancy involving the female genital tract and the fourth most common malignancy in women after breast, lung, and colorectal cancers (1). In 2012, approximately 320,000 new cases of EC were diagnosed worldwide and the incidence is increasing (2). Currently, endometrial carcinogenesis is thought to be a multi-step process involving the coordinated interaction of hormonal regulation, gene mutation, adhesion molecules, and apoptosis; however, the molecular mechanisms underlying the pathogenesis of EC have not been fully elucidated (3). Therefore, a better understanding of the molecular mechanism underlying the progression of EC will likely lead to new insights regarding novel therapeutic targets.

Hepatoma-derived growth factor (HDGF), the gene for which is located on chromosome 1q21–23, is a heparin-binding growth factor that was originally purified from media conditioned with the human hepatoma cell line, HuH7 (4). HDGF is ubiquitously expressed in normal tissues and tumor cell lines that exhibit growth factor properties. The most recent study reported that HDGF acts as a coactivator of SREBP1-mediated transcription of lipogenic genes (5). HDGF is characterized as a mitogen for many cell types and localizes to the nucleus, which is necessary for its mitogenic activity. Characteristics such as promoting growth, suppressing differentiation, and exhibiting angiogenic properties, suggest a role for HDGF in cancer induction and tumor progression (6–8). Accordingly, a number of studies have focused on the significance of HDGF as a prognostic marker and have demonstrated its clinical value for oral cancer (9), esophageal cancer (10), gastrointestinal stromal tumors (11, 12), meningiomas (13), hepatocellular cancer (14), and non-small cell lung carcinoma (15). Consistent with these findings, in our previous study (16), we determined a correlation between high nuclear expression of HDGF and clinicopathological data of EC; however, the functional significance of HDGF in EC remains unknown.

The DEAD-box RNA helicase, DDX5 (p68), constitutes a multi-functional protein with an important role in regulating transcription in conjunction with multiple, sequence-specific transcription factors (17). Recently, DDX5 has been demonstrated to act as a potent transcriptional co-activator of the estrogen receptor (18, 19), androgen receptor (20), tumor suppressor p53 (21), MyoD (22), and β-catenin (23). DDX5 has been implicated in cancer development and progression by functioning in several key cancer cell activities, such as proliferation, migration, cytoskeletal reorganization, and the epithelial-mesenchymal transition (EMT) (24–28). Recently, a small molecule inhibitor for DDX5, Supinoxin™ (RX-5902), has been developed for cancer therapy, and is currently in a clinical trial in patients with metastatic triple negative breast cancer (ClinicalTrials.gov identifier: NCT02003092) (29), which suggested the significance of DDX5 in the pathogenesis of tumor.

In the current study, we examined and characterized the interaction between HDGF and DDX5, and determined its effect on the activation of PI3K/AKT and downstream β-catenin-mediated cell cycle and EMT signaling to promote the proliferation, invasion, and metastasis of EC.

## Materials and Methods

### Cell Culture

The EC cell lines Ishikawa and RL95-2 were purchased from the Chinese Academy of Sciences Cell Bank (Shanghai, China). All cell lines were maintained in RPMI-1640 medium supplemented with 10% fetal bovine serum (ExCell, Shanghai, China). Ishikawa and RL95-2 cell lines used in this study were incubated in a humidified chamber with 5% CO_2_ at 37°C.

### Immunohistochemistry and Evaluation of Staining

One hundred and twenty two endometrial carcinoma (EC) paraffin sections (3 mm) samples from 2002 to 2008 were obtained in the Third Affiliated Hospital of Guangzhou Medical University, Guangzhou City, China. Detailed information and the IHC of HDGF about the 122 EC tissue specimens was performed in our previous study (16). Immunohistochemistry was performed according to standard procedures. The staining intensity of DDX5 (1:100, Abcam, Cambridge, MA, USA) was scored as previously described (20).

### Establishment of EC Cell Lines With Stable Knockdown of HDGF

Lentiviral particles carrying human HDGF short hairpin RNA (shHDGF-1, -2, and -3; Supplementary Table 1) vector and empty vector controls (PLV-Ctr) were constructed by GeneChem (Shanghai, China). Ishikawa and RL95-2 cells were infected with lentiviral vectors, and cells with green fluorescent protein signals (Supplementary Figure 1) were selected for further experiments using qRT-PCR and western blotting analyses.

### Transient Transfection Using Plasmids or Small Interfering RNAs or PI3K Inhibitor Ly294002

HDGF, DDX5, and β-catenin plasmids were generated by Biosense Technologies (Guangzhou, China). Small interfering RNA (siRNA) for DDX5 and β-catenin (named as siDDX5 and siβ-catenin, respectively) were designed and synthesized by Guangzhou RiboBio (RiboBio Inc., China). At 24 h before transfection, EC cells were plated onto a 6- or 96-well plate (Nest Biotech, China) at 30%–50% confluence. Plasmids were then transfected into cells using Lipofectamine™ 2,000 (Invitrogen Biotechnology, Shanghai, China), according to the manufacturer's protocol. EC cells overexpressing HDGF were treated with or without Ly294002 according to a previous description (30). Cells were collected after 48–72 h for further experiments.

### RNA Isolation, Reverse Transcription, and qRT-PCR

RNA was extracted from EC cell lines and the empty vector controls using TRIzol (TaKaRa, Shiga, Japan). RNA was transcribed into cDNA (TaKaRa) and amplified with specific primers. The targeted HDGF sequences were: sense 5′-GCT TCC GGC TAT CAG TCC TC-3′; antisense: 5′-CTG CCT CCT TCT CCT CTC CT-3′; The targeted DDX5 sense: 5′-GGC CTG ATC ACA GAA CCA TT-3′; antisense: 5′-ACC ACC CTT ATT CCC AAA CC-3′; and ARF5 (used as an internal control) sense: 5′-ATC TGT TTC ACA GTC TGG GAC G-3′; antisense: 5′-CCT GCT TGT TGG CAA ATA CC-3′. The assays were performed in accordance with the manufacturer's instructions (TaKaRa) and according to a previous description (31). The PCR reaction for each gene was repeated three times.

### Western Blotting Analysis

Western blotting was performed as previously described (32). The antibodies included anti-HDGF, DDX5, pRb, E2F1, CCND1, CDK4, c-Myc, P27, PI3K, p-PI3K, AKT, p-AKT, β-catenin, E-cadherin, N-cadherin, vimentin, Snail, and β-actin (Supplementary Table 2). β-actin was used as a loading control for all blots. The images were captured using a ChemiDoc™ CRS+ Molecular Imager (Bio-Rad, Berkeley, CA, USA).

### MTT Assay

The 3-(4, 5-dimethylthiazol-2-yl)-2, 5-diphenyltetrazolium bromide (MTT) assay was used to evaluate the rate of *in vitro* cell proliferation. For MTT assay, cells were processed as described earlier (31). Briefly, after transfected with shHDGF or PLV-Ct, cells were incubated, dissolve and measured the absorbance value (OD) at 490 nm.

### EdU Incorporation Assays

Proliferating EC cells were examined using the Cell-Light EdU Apollo 567 *in vitro* Imaging Kit (RiboBio, Guangzhou, China), according to the manufacturer's protocol. Briefly, after incubation with 10 mM EdU for 2 h, EC cells were fixed with 4% paraformaldehyde, permeabilized in 0.3% Triton X-100, and stained with Apollo fluorescent dyes. A total of 5 mg/mL of DAPI was used to stain cell nuclei for 10 min. The number of EdU-positive cells was counted under a fluorescence microscope in five random fields. All assays were independently performed three times.

### Colony Formation Assay

Cells were plated in 6-well culture plates at 200 cells/well (3 wells/cell group). After incubation at 37°C in a 5% CO_2_ incubator for 15 days, cells were washed twice with phosphate buffered saline (PBS) and stained with hematoxylin solution. The visible colony numbers were counted. All experiments were repeated at least three times.

### Cell Cycle Analysis

A total of 5 × 10^6^ EC cells were harvested after a 48-h incubation, and then washed with cold PBS. The cells were further fixed with 70% ice-cold ethanol at 4°C overnight. Fixed cells were washed three times with cold PBS. After incubation with PBS containing 10 mg/mL of propidium iodide and 0.5 mg/mL of RNase A for 15 min at 37°C. FACS caliber flow cytometry (BD Biosciences, San Jose, CA, USA) was used to obtain the DNA content of the labeled cells.

### *In vivo* Tumorigenesis in Nude Mice

The animal studies were approved by the Animal Ethics Committee of the Southern Medical University. A total of 5 × 10^6^ logarithmically growing EC cells transfected with shHDGF or PLV-Ctr (*n* = 5 per group) in 0.1 mL of RPMI-1640 medium were subcutaneously injected into the left-right symmetric flank of 4–5-week-old male BALB/c-nu mice. The mice were maintained in a barrier facility on HEPA-filtered racks. The animals were fed an autoclaved laboratory rodent diet. After 21 days, the mice were sacrificed and tumor tissues were excised and weighed.

### Wound Healing Assay

EC cells were plated in 6-well plates and incubated overnight until 90% confluent. An injury line was made using a 10-μL plastic filter tip to create a wound approximately 10 μm in diameter. Then we removed the culture medium and used PBS to eliminate dislodged cells. Subsequently, the wells were covered with serum-free medium to incubate for 48 h. “Wound closure” was observed at 0, 12, 24, 48 h under an inverted microscope.

### Transwell Migration and Invasion Assays

For cell migration assays, 1 × 10^5^ cells in 100 μL of RPMI-1640 medium without serum were seeded on a fibronectin-coated polycarbonate membrane insert in a Transwell apparatus (Corning, Armonk, NY, USA). In the lower chamber, 500 μL of RPMI-1640 with 10% serum was added as a chemoattractant. After the cells were incubated for 10 h at 37°C in a 5% CO2 atmosphere, the insert was washed with PBS and cells on the top surface of the insert were removed with a cotton swab. Cells adhering to the lower surface were fixed with methanol, stained with Giemsa solution, and counted under a microscope in 5 pre-determined fields (200×). For the cell invasion assay, the procedure was similar to the Transwell migration assay, except that the Transwell membranes were pre-coated with 24 μg/mL of Matrigel (R&D Systems, Minneapolis, MN, USA) for 4 h. All assays were independently repeated three times.

### *In vivo* Metastasis Assays

*In vivo* metastasis assays were performed according to a previous study (30). A total of 5 × 10^6^ EC-shHDGF and -PLV-Ctr cells were injected into nude mice (*n* = 5 for each group) through the liver membrane. Whole-body optical images were visualized to monitor primary tumor growth and formation of metastatic lesions. After 2 months, all mice were sacrificed, individual organs were removed, and metastatic tissues were analyzed by hematoxylin and eosin staining.

### Co-immunoprecipitation (Co-IP)

Co-IP was carried out using a Pierce Co-Immunoprecipitation kit (Thermo Scientific, Waltham, MA, USA), according to the manufacturer's instructions. The cells were lysed and the protein concentrations were measured. Then, 2000 μg of protein in 400 μL of supernatant was incubated with 10 μg anti-HDGF, anti-DDX5, and anti-β-catenin or anti-IgG antibodies coated on beads on a rotator overnight at 4°C. The beads were washed, eluted in sample buffer, and boiled for 8 min at 95°C. Immune complexes were subjected to Coomassie brilliant blue staining, mass spectrometry (Geneseed Biotech Co., Ltd, Guangzhou, China) and western blotting analysis. Anti-IgG was used as a negative control.

### Immunofluorescence

Immunofluorescent staining was performed according to standard procedures as a previous study (33). Briefly, cells were cultured overnight, then fixed with 3.5% paraformaldehyde and permeabilization by 0.2% Triton X-100 at room temperature. Cells were incubated with mouse anti-β-catenin and rabbit anti-DDX5 antibody overnight at 4°C. After three washes in PBS, the coverslips were incubated for 1 h with secondary antibody. Then, coverslips were mounted onto slides with mounting solution containing 0.2 mg/mL of DAPI and sealed with nail polish. Slides were stored in a dark box and observed using a scanning confocal microscope (Zeiss LSM 800, Oberkochen, Germany).

### Statistical Analysis

SPSS 21.0 (Chicago, IL, USA) and Graph Pad Prism 5.0 software (LaJolla, CA, USA) were used to analyze all data for statistical significance. Comparisons between two groups were performed using Student's *t*-test, one-way analysis of variance (ANOVA) for multiple groups, and a parametric generalized linear model with random effects for tumor growth and the MTT assay. Data are expressed as the mean ± SD from at least three independent experiments. The chi-squared test was applied to determine the relationship between the level of DDX5 expression and clinicopathological characteristics. Analysis of HDGF and DDX5 expression in 122 EC tissues was performed using paired-samples *t*-tests. The relationships were analyzed using Spearman's correlation analysis. Survival analysis was performed using the Kaplan-Meier method. The multivariate Cox proportional hazards method was used for analyzing the relationship between variables and patient survival time. A prognostic value of < 0.05 (*P* < 0.05) was considered significant, and all tests were two-sided. Statistical significance was denoted as follows: ^*^P < 0.05, ^**^P < 0.01, ^***^P < 0.001.

## Results

### Stable Knockdown of HDGF Expression Inhibits Cell Proliferation *in vitro* and *in vivo*

**Figure 1:**
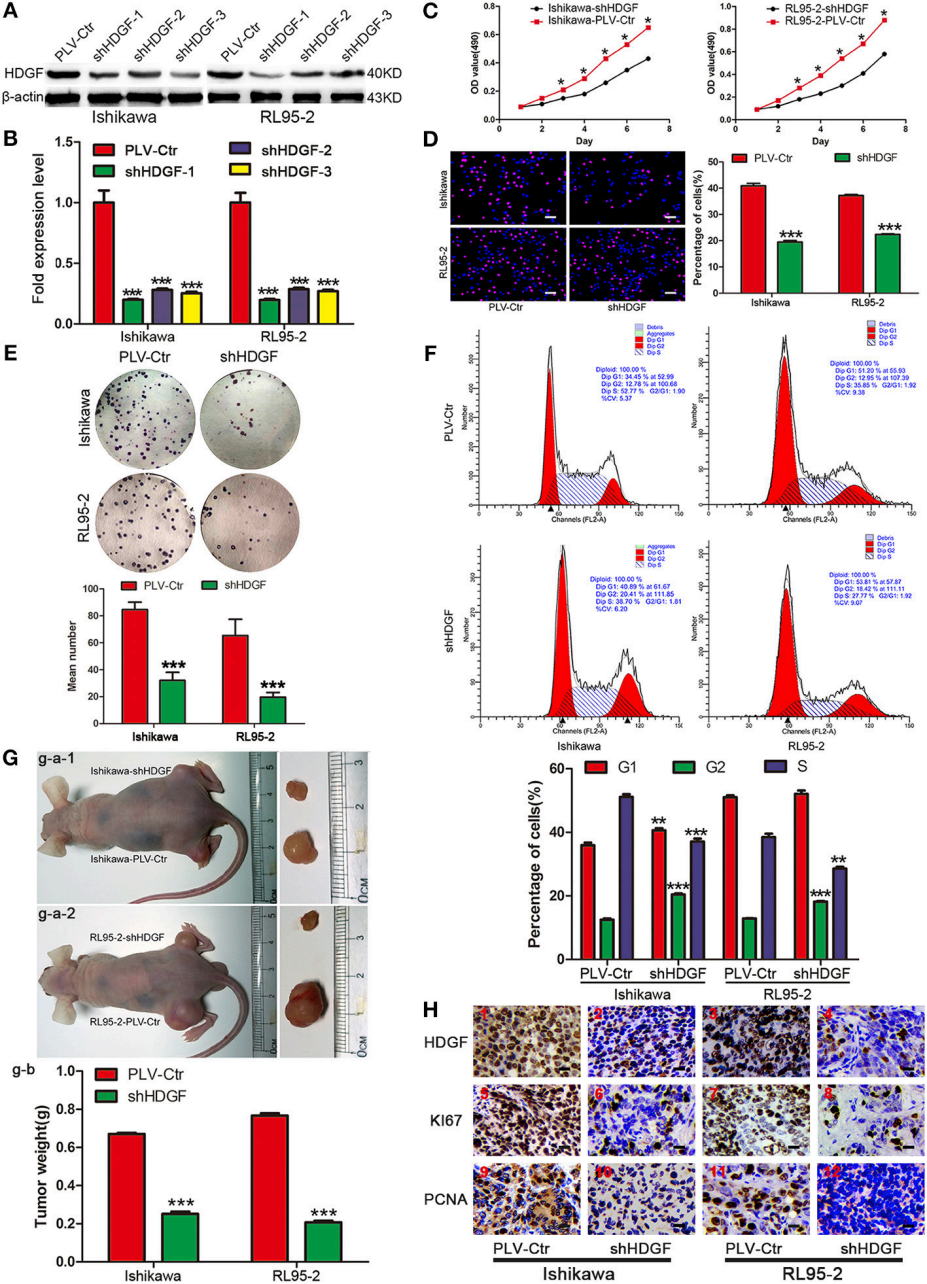
Stable knockdown HDGF inhibits cell growth in EC. HDGF knockdown confirmed by western blot **(A)** and qRT-PCR **(B)**. Student's *t*-test, ***P < 0.001. MTT assay **(C)**, EdU incorporation assays **(D)**, clone formation **(E)**, and cell cycle assay **(F)** after HDGF knockdown. One-way ANOVA and Dunnett's multiple comparison test, *P < 0.05. **(G)** g–a: When compared with PLV-Ctr, *in vivo* tumorigenicity of shHDGF-EC cells was markedly reduced (***P < 0.001). g–b: Tumor weight statistics for each mouse group (*n* = 5 per group) **(H)**. HDGF, Ki67, and PCNA was evaluated by immunohistochemical. Compared with shHDGF-EC cells (2,4,6,8,10,12), the PLV-Ctr cell tumor tissues(1,3,5,7,9,11) were high expression.

To gain insight into the role of HDGF in EC, we first used a lentiviral vector to specifically and stably knockdown the expression of HDGF in the Ishikawa and RL95-2 cells. The levels of HDGF were assessed by western blotting (Figure 1A) and qRT-PCR (Figure 1B). The most efficient knockdowns of HDGF expression were shown in shHDGF-3-Ishikawa and shHDGF-1-RL95-2 cells compared to the PLV-Ctr (*P* < 0.001) (Supplementary Figure 2).

**Figure 2:**
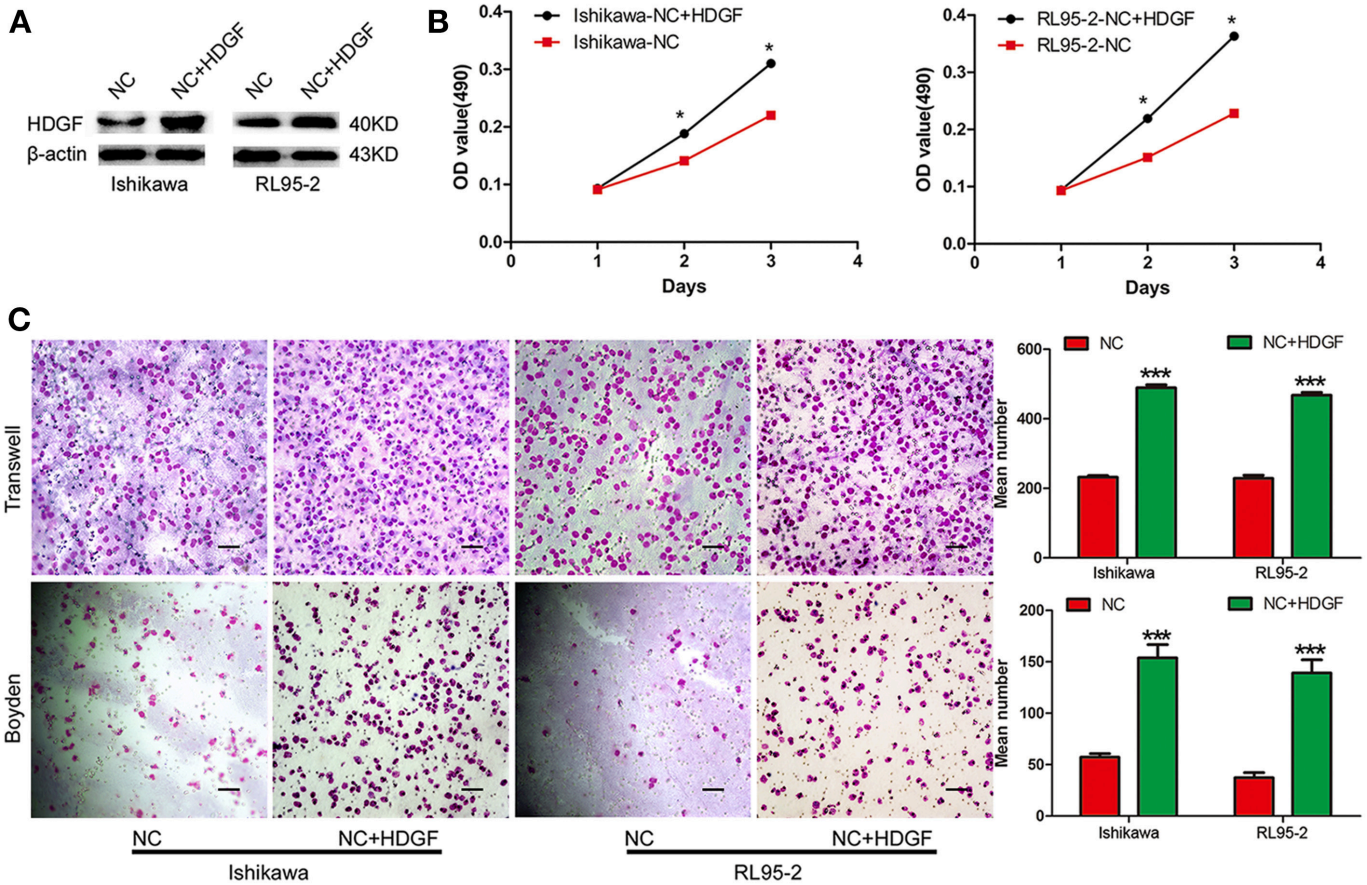
Transient overexpression of HDGF by plasmid-promoted cell proliferation, migration, and invasion. Efficiency of plasmid overexpression of HDGF in EC cell lines **(A)**. Transient increase in expression of HDGF by plasmid promoted cell proliferation in EC cells **(B)**. Transient upregulation of HDGF dramatically increased the migration and invasion ability of EC cells *in vitro* **(C)**. Data are presented as the mean ± SD for three independent experiments (*P < 0.05, ***P < 0.001).

Next, we assessed the effect of decreased HDGF expression on EC cell growth *in vitro*. The growth curves determined by MTT assays showed that growth of the shHDGF-Ishikawa and shHDGF-RL95-2 cells was significantly slower than that of PLV-Ctr cells (*P* < 0.05; Figure 1C). Conversely, overexpression of HDGF (Figure 2A) reversed these effects (*P* < 0.05; Figure 2B). The EdU incorporation assay revealed that the percentage of cells in S phase decreased following the downregulation of HDGF expression (*P* < 0.001; Figure 1D). Colony formation assays showed that knockdown of HDGF significantly decreased cell proliferation (*P* < 0.001; Figure 1E). Furthermore, cell cycle analysis demonstrated that HDGF suppression dramatically reduced cell cycle progression from the G1 to S phase (*P* < 0.05; Figure 1F).

Subsequently, to confirm the growth effect of HDGF *in vivo*, we performed an *in vivo* tumorigenesis study by inoculating EC cells into nude mice. Mice in the shHDGF-EC and PLV-Ctr groups were sacrificed 21 days after inoculation, with average tumor weights of 0.187 g and 0.793 g, respectively (*P* < 0.001; Figure 1G). The mice injected with shHDGF-Ishikawa and shHDGF-RL95-2 cells had smaller tumor burdens (Figure 1G) and displayed lower expression of HDGF, Ki67 and proliferating cell nuclear antigen (PCNA) in tumor tissues relative to the controls (Figure 1H). These results suggested that HDGF significantly promotes tumorigenesis *in vivo*.

### HDGF Downregulation Suppresses Cell Migration, Invasion, and Intrahepatic Metastasis of EC Cells *in vitro* and *in vivo*

**Figure 3:**
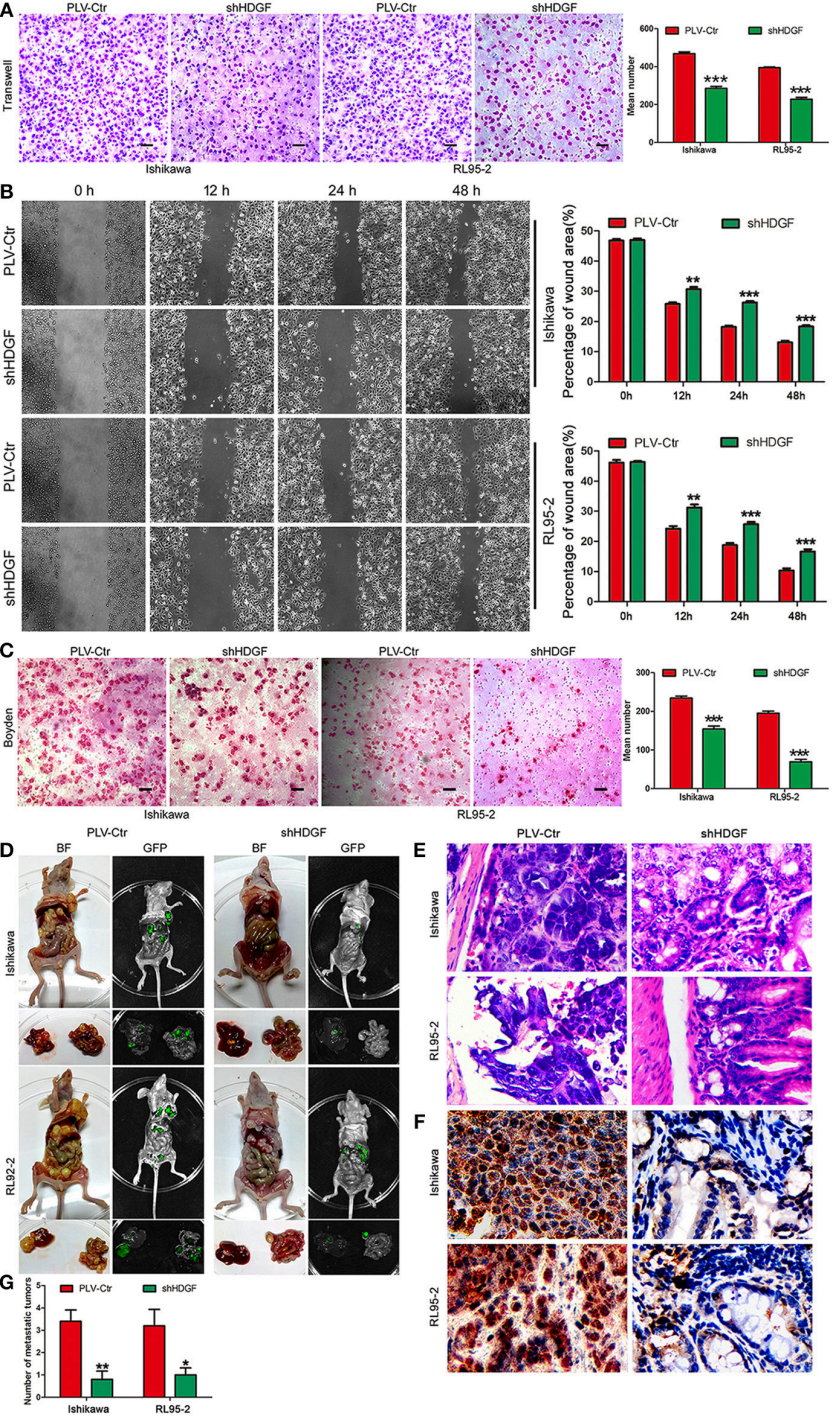
HDGF expression knockdown decreases cell migration, invasion, and metastasis. Stable downregulation of HDGF reduced the *in vitro* migration ability of Ishikawa and RL95-2 cells **(A)**. The wound healing assay indicated that shHDGF transfection into Ishikawa and RL95-2 cells for 48 h impaired migration capacity compared with the negative control group **(B)**. Stable suppression of HDGF reduced the *in vitro* invasiveness of shHDGF-Ishikawa and shHDGF-RL95-2 cells **(C)**. *In vivo* intrahepatic metastasis assay results after shHDGF-Ishikawa and PLV-Ctr-Ishikawa injection: PLV-Ctr-EC cells were more easily transferred to intestinal tissue **(D)**. The HE **(E)** and IHC **(F)** of the metastatic intestinal tissues. Original magnification 200×. The numbers of metastatic tumors **(G)**. Data are presented as the mean ± SD for three independent experiments. ^*^P < 0.05, ^**^P < 0.01, ^***^P < 0.001, statistically significant difference.

To examine the effect of HDGF on cell migration and invasion, a Transwell apparatus, wound healing assay, and Boyden chamber coated with Matrigel were used. After incubation for 10 h, a reduced number of migrated cells were observed for shHDGF-Ishikawa and shHDGF-RL9-2 cells compared with PLV-Ctr cells (*P* < 0.001; Figure 3A). In addition, the wound healing assay demonstrated that shHDGF-Ishikawa and shHDGF-RL95-2 cells inhibited the migration capacity (*P* < 0.05; Figure 3B). As shown in Figure 3C, the results of the Boyden chamber coated with Matrigel assays were similar to those of the Transwell assays (*P* < 0.01 for each); however, overexpressing HDGF reversed these effects (*P* < 0.05; Figure 2C).

To further assess the effect of HDGF on EC intrahepatic metastasis, shHDGF-Ishikawa and control cells were independently injected into the liver capsules of nude mice. Fluorescence imaging was used to identify scattered metastatic nodules in the livers and intestines of nude mice that formed in the mice after 2 months. Only a few scattered metastatic cells were observed following injection of shHDGF-Ishikawa cells, whereas a variety of large clusters were observed in the PLV-Ctr group (Figure 3D). As can be seen from Figure 3D, control PLV-Ctr cells were more easily transferred to intestinal tissue, while shHDGF was less or not metastasized. The HE and IHC of the metastatic intestinal tissues and numbers of metastatic foci were shown in Figures 3E–G. Taken together, these results suggest that HDGF effectively promotes cell migration, invasion, and intrahepatic metastasis of EC cells *in vitro* and *in vivo*.

### HDGF Regulates the Expression of Cell Cycle- and EMT-Associated Genes via the PI3K/AKT and β-Catenin Signaling Pathways in EC Cells

**Figure 4:**
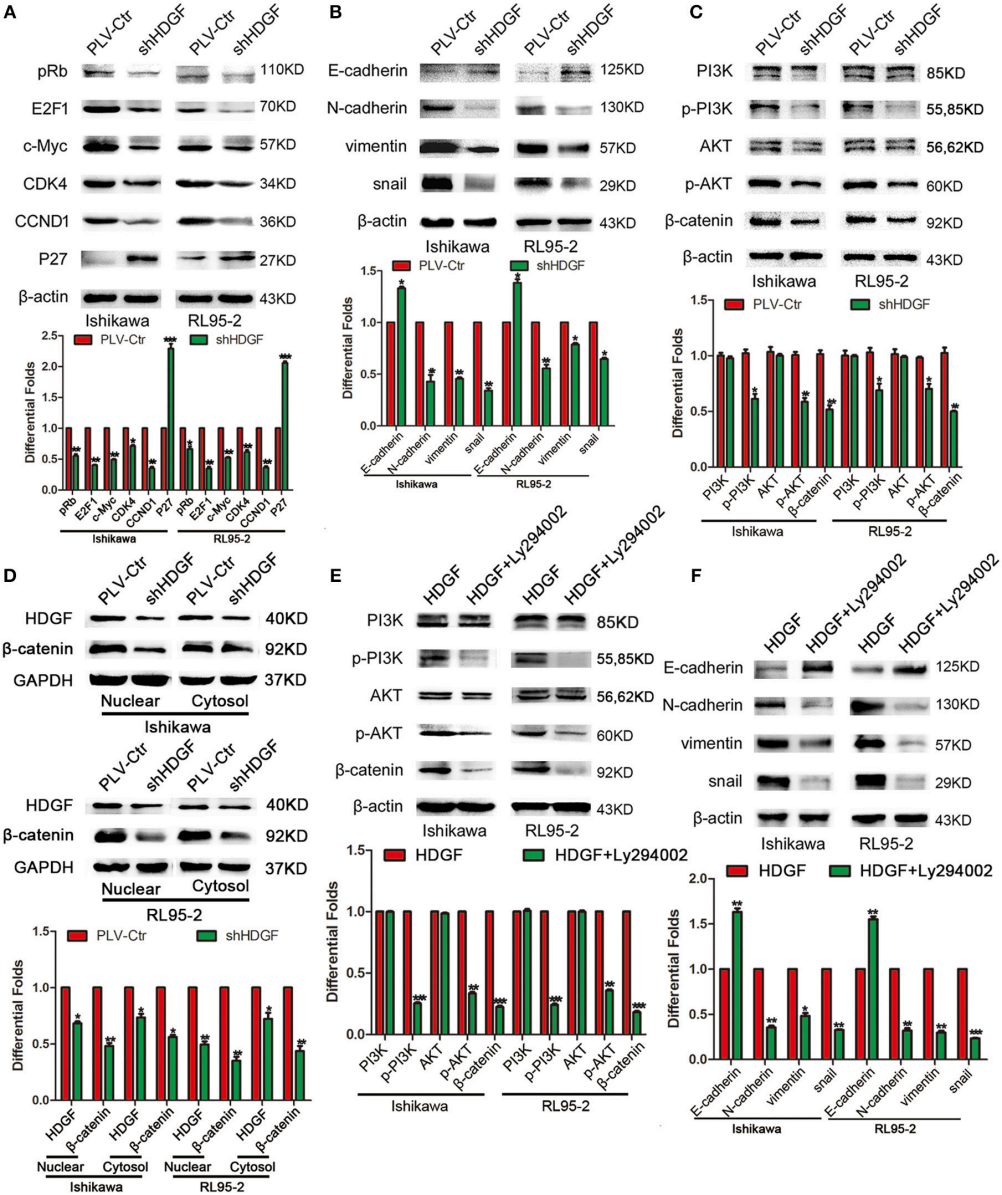
HDGF controls cell cycle and EMT-associated gene expression in EC via the PI3K/AKT and β-catenin pathways. Knocking down endogenous HDGF expression reduced the expressions of pRb, E2F1, c-Myc, CDK4, and CCND1, and enhanced P27 in EC cells **(A)**. Suppressing HDGF expression decreased the expression of EMT marker genes (N-cadherin, Vimentin, and Snail) and enhanced E-cadherin expression **(B)**. Reduced HDGF expression decreased the expressions of p-PI3K, p-AKT, and β-catenin **(C)**. Knocking down of HDGF suppressed both nuclear and cytosol protein expression of β-catenin **(D)**. Suppressing the expression of p-PI3K by its specific inhibitor Ly294002 (50 nM) reduced p-AKT, β-catenin, Snail, N-cadherin, Vimentin, and upregulated E-cadherin in overexpressing HDGF EC cells **(E,F)**. β-actin served as the internal control. Each experiment was repeated three times with similar results, and error bars represent the mean ± SD, *P < 0.05, **P < 0.01, ***P < 0.001.

To further study the mechanism by which HDGF regulates cell proliferation, migration, invasion, and metastasis, the protein levels of cell cycle- and EMT-associated genes were examined in Ishikawa and RL95-2 cells with stably suppressed HDGF. Knockdown of HDGF inhibited the activation of oncogenic cell cycle regulators, including pRb, E2F1, c-Myc, CCND1, and CDK4, and increased the level of P27 (Figure 4A). Further, we found that EMT markers (N-cadherin, Vimentin, and Snail) were suppressed, whereas the E-cadherin level increased (Figure 4B). Simultaneously, the levels of p-PI3K, p-AKT, and β-catenin were significantly decreased (Figure 4C). Further, we observed that knockdown of HDGF significantly suppressed both nuclear and cytosol protein expressions of β-catenin in EC cells (Figure 4D). In subsequent study, we used specific inhibitor (Ly294002) of PI3K to suppress the expression of p-PI3K and observed that the protein expression of p-PI3k, p-AKT, β-catenin, Snail, N-cadherin, Vimentin was decreased, and E-cadherin was upregulated in overexpressing HDGF EC cells (Figures 4E,F). These results suggest that HDGF regulates the expression of cell cycle- and EMT-associated genes via the PI3K/AKT and β-catenin signaling pathways in EC cells.

### DDX5 Interacts With HDGF and β-Catenin

**Figure 5:**
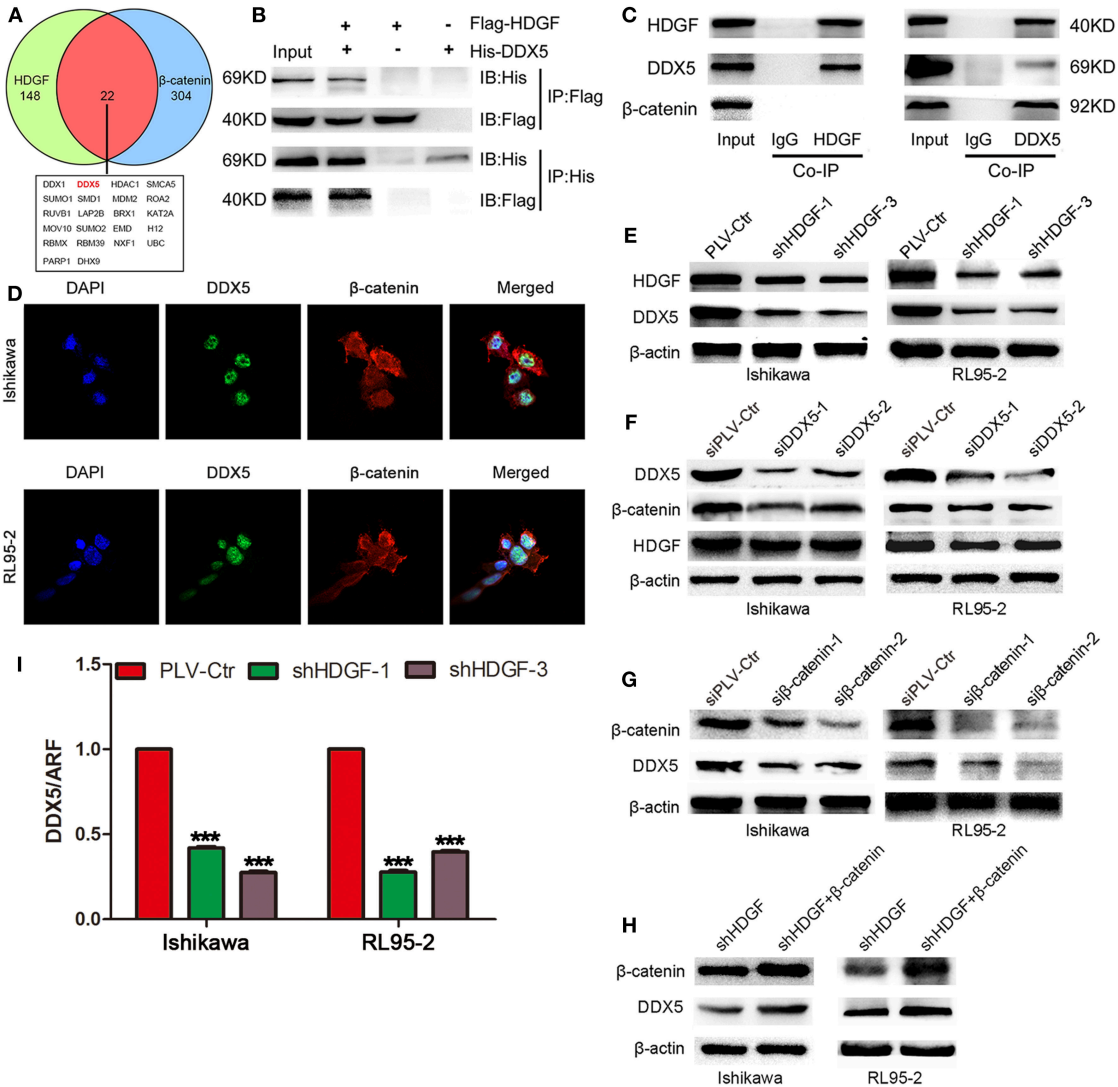
DDX5 interact with both HDGF and β-catenin in EC cells. Venn diagram depicting overlaps between HDGF and β-catenin proteins: the 22 common proteins are listed **(A)**. Co-immunoprecipitation (Co-IP) detected the interaction of exogenous HDGF and DDX5 in RL95-2 cells **(B)**. Co-IP detected the interaction of endogenous HDGF and DDX5 in RL95-2 cells **(C)**. Nuclear co-localization of HDGF and DDX5, as well as β-catenin and DDX5 proteins, in EC cells by immunofluorescence under a scanning confocal microscope **(D)**. DDX5 levels after HDGF knockdown, as assessed by western blotting **(E)**. HDGF and β-catenin levels after DDX5 knockdown **(F)**. Overexpression of β-catenin plasmid in shHDGF-EC cells increased DDX5 levels **(G)**. DDX5 expression after β-catenin knockdown **(H)**. DDX5 mRNA expression after HDGF knockdown, normalized to the expression of ARF5. One-way ANOVA and Dunnett's multiple comparison test ***P < 0.001 **(I)**.

In previous study, our team had shown the interaction of β-catenin and HDGF or DDX5 in Lung Adenocarcinoma (33). To explore the precise molecular mechanisms of HDGF in EC, co-IP, combined with mass spectrometry, was used in Ishikawa cells. This analysis yielded 242 potential HDGF-interacting proteins (Supplementary Table 3), including DDX5 (69 kDa band) and β-catenin (92 kDa band). In addition, we used data sets from public domain data to draw a Venn diagram to show the proteins that interact with HDGF (34) and β-catenin (35) proteins, and observed that there were 22 overlapping proteins (Figure 5A). Exogenous and endogenous co-IP demonstrated that HDGF and DDX5 interact in Ishikawa cells, whereas β-catenin was associated with DDX5, but not HDGF (Figures 5B,C). Moreover, nuclear co-localization of DDX5 and β-catenin proteins, as well as that of HDGF and DDX5 proteins (33), was observed by immunofluorescence using a scanning confocal microscope (Figure 5D). Taken together, these results suggest that HDGF is associated with DDX5 in EC, as is the case with β-catenin and DDX5.

Subsequently, western blotting indicated that stable knockdown of HDGF expression resulted in DDX5 protein downregulation (Figure 5E); however, knockdown of DDX5 did not significantly affect HDGF expression, but resulted in β-catenin protein downregulation (Figure 5F). In addition, we observed that β-catenin suppression decreased the level of the DDX5 protein (Figure 5G). Furthermore, overexpression of β-catenin from a plasmid in shHDGF-EC cells increased DDX5 expression (Figure 5H). qRT-PCR showed that the expression of DDX5 was reduced after knockdown of HDGF (Figure 5I), which indicated that HDGF affects DDX5 at the level of transcription.

### DDX5 Overexpression Reverses the Suppression of shHDGF

**Figure 6:**
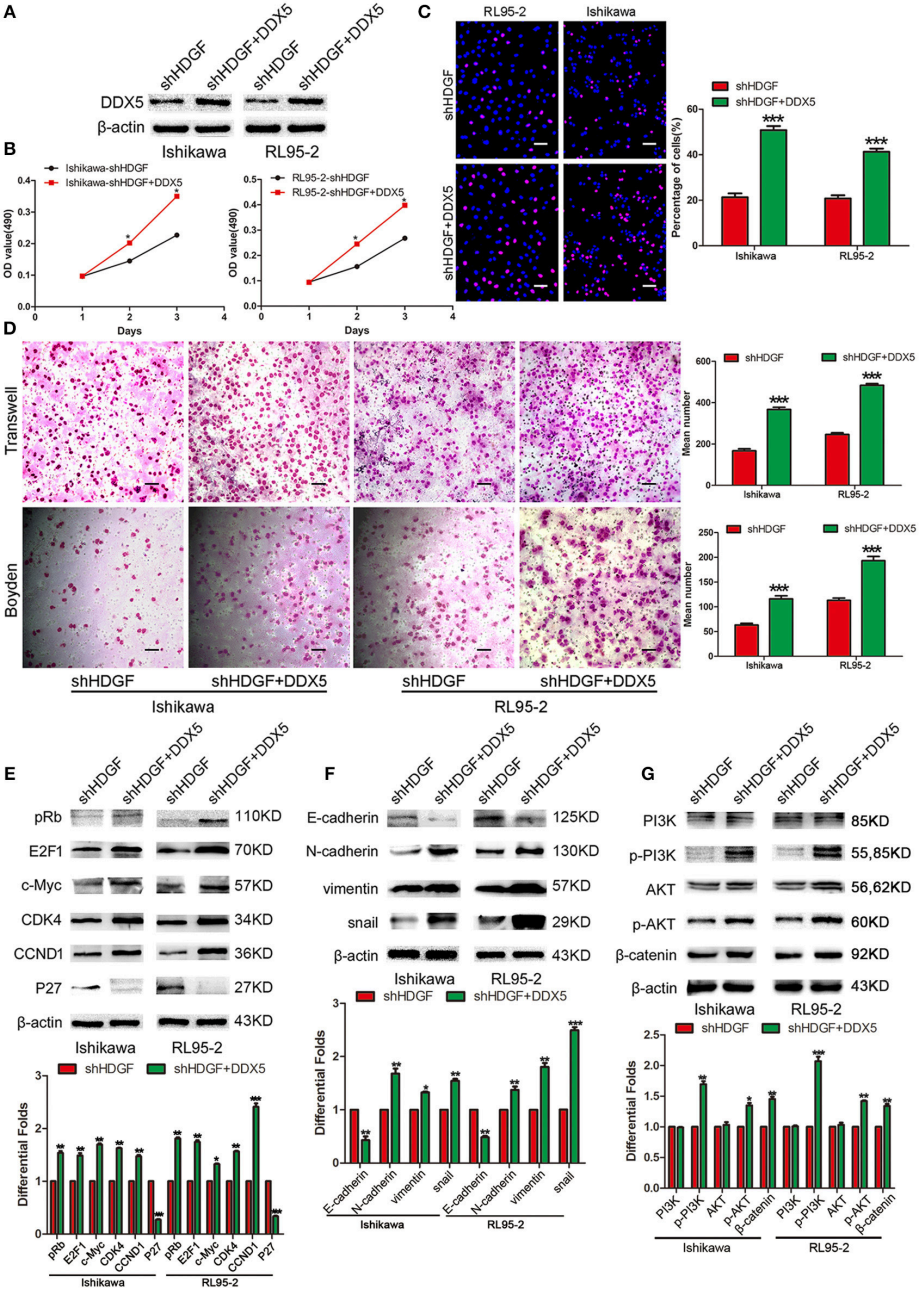
Transient overexpression of DDX5 from a plasmid reverses the suppression of shHDGF. Efficiency of plasmid overexpression of DDX5 in EC cell lines **(A)**. Transient increase in the expression of DDX5 by plasmid promotes cell proliferation in EC cells, as assessed by the MTT **(B)** and EdU incorporation assays **(C)**. Transient upregulation of DDX5 dramatically increases the migration and invasion ability of EC cells *in vitro* **(D)**. Western blotting analysis of the protein levels of p-PI3K, p-AKT, β-catenin, pRb, E2F1, c-Myc, CDK4, CCND1, and P27, as well as invasion and migration according to the relevant protein levels of E-cadherin, N-cadherin, Vimentin, and Snail after transient transfection of DDX5 plasmid into EC cells **(E–G)**. β-actin served as the internal control. Data are presented as the mean ± SD for three independent experiments (*P < 0.05, **P < 0.01, ***P < 0.001).

Transiently transfecting DDX5 into shHDGF EC cells (Figure 6A) enhanced cell proliferation, as assessed by the MTT (Figure 6B) and EdU incorporation assays (Figure 6C). Transwell and Boyden chamber assays showed that EC cell migration and invasive ability were enhanced (Figure 6D). Furthermore, we found that DDX5 overexpression induced the expression of pRb, E2F1, c-Myc, CCND1, CDK4, N-cadherin, Vimentin, Snail, p-PI3K, p-AKT, and β-catenin, but reduced the expression of P27 and E-cadherin (Figures 6E–G). These results indicated that DDX5 overexpression can overcome the EC cell growth suppression induced by shHDGF.

### Association of HDGF and DDX5 Expression With the Clinicopathological Characteristics of EC Tissues

**Figure 7:**
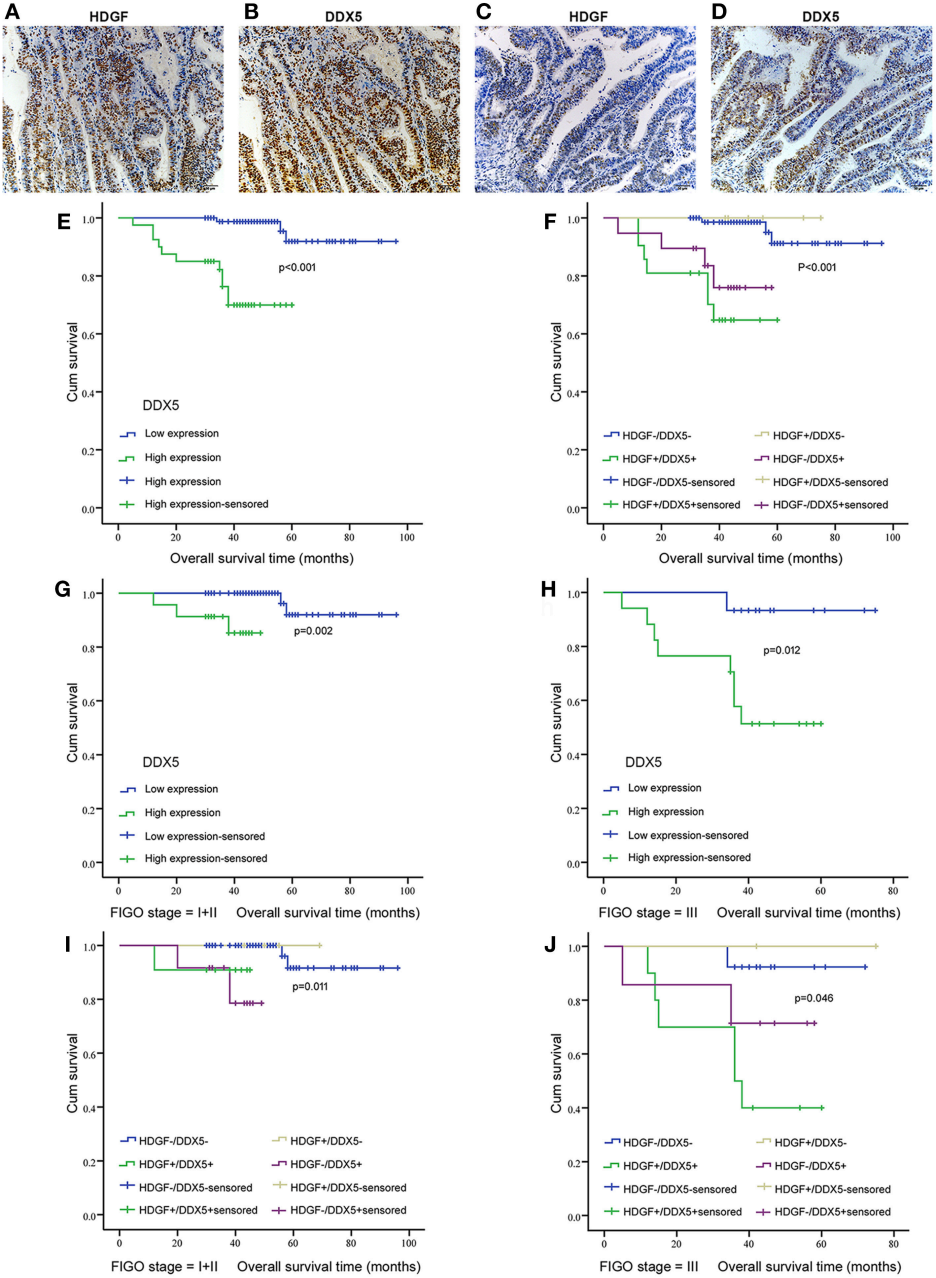
Correlation between HDGF and DDX5 in EC tissues and the clinical significance. Immunohistochemistry results showing the level of HDGF and DDX5 expression at the same locations in EC tissues. **(A,B)** represent high expression of HDGF and DDX5, respectively. **(C,D)** represent low expression of HDGF and DDX5, respectively. Original magnification 200×. We analyzed DDX5 expression in the primary tumors of patients with EC **(E)** and the correlation between HDGF high expression and prognosis for patients with EC by strata analysis against FIGO stage **(G,H)**. Kaplan-Meier survival curve comparing subgroups based on HDGF and DDX5 expression in the primary tumors of patients with EC. Low HDGF expression cases with high or low DDX5 expression, as well as high HDGF expression cases with high or low DDX5 expression **(F–J)**. The *P*-value is based on a log-rank test.

Combined with data from our previous work (16), Figures 7A–D displays the expression of HDGF and DDX5. Immunohistochemical staining showed that HDGF and DDX5 positive signals were mostly located in the nuclei of EC cells, with minor cytoplasmic distribution. Our previous results indicated that 25.5% (31/122) and 74.5% (91/122) of cases exhibited high and low nuclear expression of HDGF, respectively (16). Here, we found that DDX5 protein was positively expressed in 32.8% (40/122) and not expressed in 67.2% of tumors (82/122; Table 1). DDX5 expression was significantly related to FIGO stage (I+II vs. III; *P* < 0.001; Table 1); however, no significant correlation existed with respect to age, menopausal status, histologic grading, depth of myometrial invasion, or lymph node status in patients with EC (*P* > 0.05; Table 1). The results were similar to those obtained for the HDGF protein.

**Table 1:** Correlation between the clinicopathological factors and the expression of HDGF and DDX5 in endometrial cancer.

	**HDGF (%)**				**DDX5 (%)**			
**Characteristics**	***N***	**High**	**Low**	***P***	***N***	**High**	**Low**	***P***
**Age**								
< 50	42	10(23.8)	32(76.2)	0.830	42	13(31.0)	29(69.0)	0.754
≧50	80	21(26.3)	59(73.7)		80	27(33.8)	53(66.2)	
**Menopausal status**								
Premenopausal	65	15(23.1)	50(76.9)	0.537	65	18(27.7)	47(72.3)	0.201
Postmenopausal	57	16(28.1)	41(71.9)		57	22(38.6)	35(61.4)	
**FIGO stage**								
I+II	90	18(20.0)	72(80.0)	0.032	90	20(22.2)	70(77.8)	<0.001
III	32	13(40.6)	19(59.4)		32	20(62.5)	12(37.5)	
**Histological grading**								
G1	44	15(34.1)	29(65.9)	0.140	44	15(34.1)	29(65.9)	0.067
G2	62	11(17.7)	51(82.3)		62	16(25.8)	46(74.2)	
G3	16	5(31.3)	11(68.7)		16	9(56.3)	7(43.7)	
**Depthof myometrial invasion**								
< 50%	85	19(22.4)	66(77.6)	0.263	85	27(31.8)	58(68.2)	0.834
≧50%	37	12(32.4)	25(67.6)		37	13(35.1)	24(64.9)	
**Lymph node status**								
Negative	105	25(23.8)	80(76.2)	0.369	105	32(30.5)	73(69.5)	0.264
Positive	17	6(35.3)	11(64.7)		17	8(47.1)	9(52.9)	

### Correlation Analysis of HDGF and DDX5 With Overall Survival of Patients With EC

We stratified the data from patients with EC into four groups, according to the combination of different levels of HDGF and DDX5 expression: low HDGF/low DDX5 (HDGF–/DDX5–) expression; high HDGF/high DDX5 (HDGF+/DDX5+) expression; low HDGF/high DDX5 (HDGF–/DDX5+) expression; and high HDGF/high DDX5 (HDGF+/DDX5+) expression. Our previous work showed that 25.5% (31/122) and 74.5% (91/122) of cases exhibited high and low nuclear expression of HDGF, respectively (16). Here, we found that 22 EC tissues were HDGF+/DDX5+, whereas 73 cases were HDGF–/DDX5– (Table 2). The expression of HDGF+/DDX5+ correlated with the FIGO stage (*P* = 0.003; Table 2).

**Table 2:** Co-expression of HDGF and DDX5 in endometrial cancer.

**Characteristics**	**N**	**HDGF and DDX5(%)**				***P***
		**HDGF+/DDX5+**	**HDGF+/DDX5-**	**HDGF–/DDX5+**	**HDGF–/DDX5–**	
Age						0.988
< 50	42	7(16.7)	3(7.1)	6(14.3)	26(61.9)	
≧50	80	15(18.8)	6(7.5)	12(15.0)	47(58.8)	
Menopausal status						0.647
Premenopausal	65	10(15.4)	5(7.7)	8(12.3)	42(64.6)	
Postmenopausal	57	12(21.1)	4(7.0)	10(17.5)	31(54.4)	
FIGO stage						0.003
I+II	90	10(11.1)	8(8.9)	10(11.1)	62(68.9)	
III	32	12(37.5)	1(3.1)	8(25.0)	11(34.4)	
Histological grading						0.130
G1	44	11(25.0)	4(9.1)	4(9.1)	25(56.8)	
G2	62	6(9.7)	5(8.1)	10(16.1)	41(66.1)	
G3	16	5(31.3)	0(0)	4(25.0)	7(43.8)	
Depth of myometrial invasion						0.605
< 50%	85	13(15.3)	6(7.1)	14(16.5)	52(61.2)	
≧50%	37	9(24.3)	3(8.1)	4(10.8)	21(56.8)	
Lymph node status						0.546
Negative	105	17(16.2)	8(7.6)	15(14.3)	65(61.9)	
Positive	17	5(29.4)	1(5.9)	3(17.6)	8(47.1)	

Patients with high expression of DDX5 had worse prognoses than those with low expression of DDX5 (*P* < 0.001; Figure 7E), and HDGF+/DDX5+ also correlated with a short survival time for patients with EC (*P* < 0.001; Figure 7F). We further conducted survival analysis by strata analysis against FIGO stage. These results indicated that high DDX5 protein levels and HDGF+/DDX5+ are significantly associated with the survival time for patients with EC based on FIGO stage = I+II (*P* = 0.002, *P* = 0.011, respectively; Figures 7G,I) and FIGO stage = III (*P* = 0.012, *P* = 0.046, respectively; Figures 7H,J).

Univariate analyses showed that FIGO stage, histological grading, lymph node status, depth of myometrial invasion, high DDX5 expression, and post-operative hormone therapy significantly correlated with patient survival (*P* = 0.002, *P* = 0.001, *P* < 0.001, *P* < 0.001, *P* < 0.001, and *P* = 0.044, respectively; Table 3). Multivariate analysis showed that the level of DDX5 expression, FIGO stage, and depth of myometrial invasion were independent prognostic factors for EC (*P* = 0.038, *P* = 0.018, and *P* = 0.014, respectively; Table 3). However, HDGF expression, histological grading, lymph node status, and post-operative chemotherapy were not independent prognostic factors for EC (*P* = 0.984, *P* = 0.160, *P* = 0.702, and *P* = 0.631, respectively; Table 3).

**Table 3:** Summary of univariate and multivariate Cox regression analysis of overall survival duration.

**Parameter**	**Univariate analysis**			**Multivariate analysis**		
	***P***	**HR**	**95% CI**	***P***	**HR**	**95% CI**
**Age**						
< 50 vs. ≧50	0.093	0.401	0.138–1.165			
**Family history of tumor**						
Negative vs. positive	0.279	0.325	0.043–2.487			
**Education**						
< Graduation vs. ≧graduation	0.298	26.921	0.05–13271.753			
**Health insurance**						
No vs. yes	0.089	0.020	0.000–1.811			
**Career**						
≦Worker vs. >worker	0.272	27.978	0.07–10713.674			
**Menopausal status**						
Premenopausal vs. postmenopausal	0.559	0.721	0.240–2.160			
**Complications**						
With vs. without	0.125	0.309	0.069–1.384			
**FIGO stags**						
I +II vs. III	0.002	5.652	1.892–16.883	0.018	4.385	1.283–14.994
**Histological grading**						
G1 vs. G2 vs. G3	0.001	4.514	1.896–10.745	0.160	2.178	0.735–6.453
**Lymph node status**						
Negative vs. positive	<0.001	12.232	4.196–35.659	0.702	1.477	0.200–10.905
**Depth of myometrial invasion**						
< 50% vs. ≧50%	<0.001	9.745	2.713–34.999	0.014	7.867	1.522–40.678
**HDGF expression**						
Low vs. high	0.004	4.951	1.686–14.544	0.984	0.985	0.245–3.964
**DDX5 expression**						
Low vs. high	<0.001	12.126	3.209–45.821	0.038	5.677	1.100–29.281
**Postoperative irradiation**						
Yes vs. no	0.512	1.652	0.368–7.409			
**Postoperative chemotherapy**						
Yes vs. no	0.175	2.081	0.721–6.005			
**Postoperative hormone therapy**						
Yes vs. no	0.044	0.267	0.074–0.963	0.631	1.491	0.293–7.597

### Correlation Between HDGF and DDX5 Expression in EC

There was a significant positive correlation between HDGF and DDX5 protein levels in EC tissues (*r* = 0.475, *P* < 0.001; Table 4).

**Table 4:** Correlation between HDGF and DDX5 expression in endometrial cancer tissues.

	**HDGF**		***r***	***p***
	**High**	**Low**		
DDX5			0.475	<0.001
High	22	18		
Low	9	73		

## Discussion

In previous studies, HDGF has been shown to promote the progression of tumors by activating the AKT–MAPK (36), Akt and TGF-β (37), and VEGF signaling pathways (38), and interacting with β-catenin as a positive feedback loop (39); however, the molecular mechanism involved in HDGF-associated EC cell proliferation, invasion, and metastasis has not been elucidated.

In the present study, we observed that HDGF knockdown markedly decreased cell proliferation, migration, invasion, and metastasis *in vitro*. Furthermore, subcutaneous tumor experiments in nude mice demonstrated that knockdown of HDGF inhibited tumorigenesis, invasion, and metastasis *in vivo*. These findings are consistent with an earlier report by Zhou et al. (40) in which downregulation of HDGF inhibited the proliferation and invasiveness of hepatocellular carcinoma cells.

Multiple studies have shown that PI3K/AKT constitutes a key signal mediator during carcinogenesis (41, 42) and that activation of PI3K/AKT may regulate β-catenin signaling through Akt phosphorylation and inactivation of GSK3-β (43). In the current study, we observed that decreased HDGF expression suppressed p-PI3K and p-AKT levels, and the downstream β-catenin-mediated cell cycle and EMT signal molecules, such as pRb, E2F1, c-Myc, CCND1, CDK4, Snail, Vimentin, and N-cadherin, while elevating the expression of P27 and E-cadherin in EC cells. In addition, using Ly294002 to treat overexpressing HDGF EC cells induced a decrease of p-PI3K, p-AKT, β-catenin and metastatic effect related proteins such as Snail, Vimentin, and N-cadherin. Therefore, we hypothesized that HDGF knockdown significantly suppresses EC cell proliferation, migration, invasion, and metastasis through the PI3K/AKT and β-catenin pathways in EC.

To better understand the molecular mechanisms underlying HDGF promotion of EC proliferation and metastasis, we searched public domain data and screened DDX5 as a candidate interaction protein of HDGF. DDX5 is a member of DEAD box family proteins that plays an important role in the progression of many tumors (44–48). Subsequently, we observed that HDGF not only combined with DDX5 but also induced the expression of DDX5 in EC. Furthermore, DDX5 could reverse the inhibitive effects on cell growth, migration, and invasion in shHDGF EC cells. Previously, HDGF was found to bind the promoter of β-catenin, resulting in β-catenin transcriptional activation (39). Phosphorylated p68 (DDX5) can enter the cytoplasm, which leads to its interaction with β-catenin and displacement of Axin (49). DDX5 (P68) forms a complex with β-catenin and facilitates its transcription activation to regulate both cell adhesion and gene expression (23). Notably, as β-catenin can, in turn, function as a transcription factor to stimulate DDX5 expression by binding to its promoter (50), we thus speculated that HDGF upregulated the expression of DDX5 by inducing β-catenin expression. Consistent with this speculation, we observed that DDX5 was significantly increased in shHDGF-EC cells after transfection of the β-catenin cDNA.

In previous reports, DDX5 had been documented to promote cell proliferation and EMT by interacting with Wnt-β-catenin signaling and inducing the nuclear translocation of β-catenin (23, 26, 28, 51). Consistent with these previous reports, we observed that DDX5 not only directly combined with β-catenin, but also induced the expression of β-catenin via activating PI3K/AKT signaling and stimulated its nuclear translocation, thus inducing cell cycle transition and EMT signaling and the promotion of cell growth and metastasis in EC. These data demonstrated that HDGF interacts with DDX5 to further induce β-catenin to participate in cell growth and metastasis in EC.

In our previous study of HDGF expression in 122 samples of EC, we found that high nuclear expression of HDGF was positively correlated with FIGO stage (*P* = 0.032) and that patients with high expression of HDGF had poorer overall survival rates compared to those with low expression (*P* = 0.001) (16). In the current study, we further showed that DDX5 protein was expressed in 32.8% (40/122) of EC samples. Similar to HDGF, DDX5 expression was also significantly associated with FIGO stage (*P* < 0.001), and patients with high expression of DDX5 had poorer overall survival rates than those with low expression (*P* < 0.001). Multivariate analyses showed that high expression of DDX5 was an independent predictor of prognosis for patients with EC (*P* = 0.038). Notably, our data showed a significant positive correlation between HDGF and DDX5 in EC tissues (*r* = 0.475, *P* < 0.001). Finally, we observed that high nuclear levels of HDGF and DDX5 led to the worst prognosis for patients with EC.

## Conclusions

**Figure 8:**
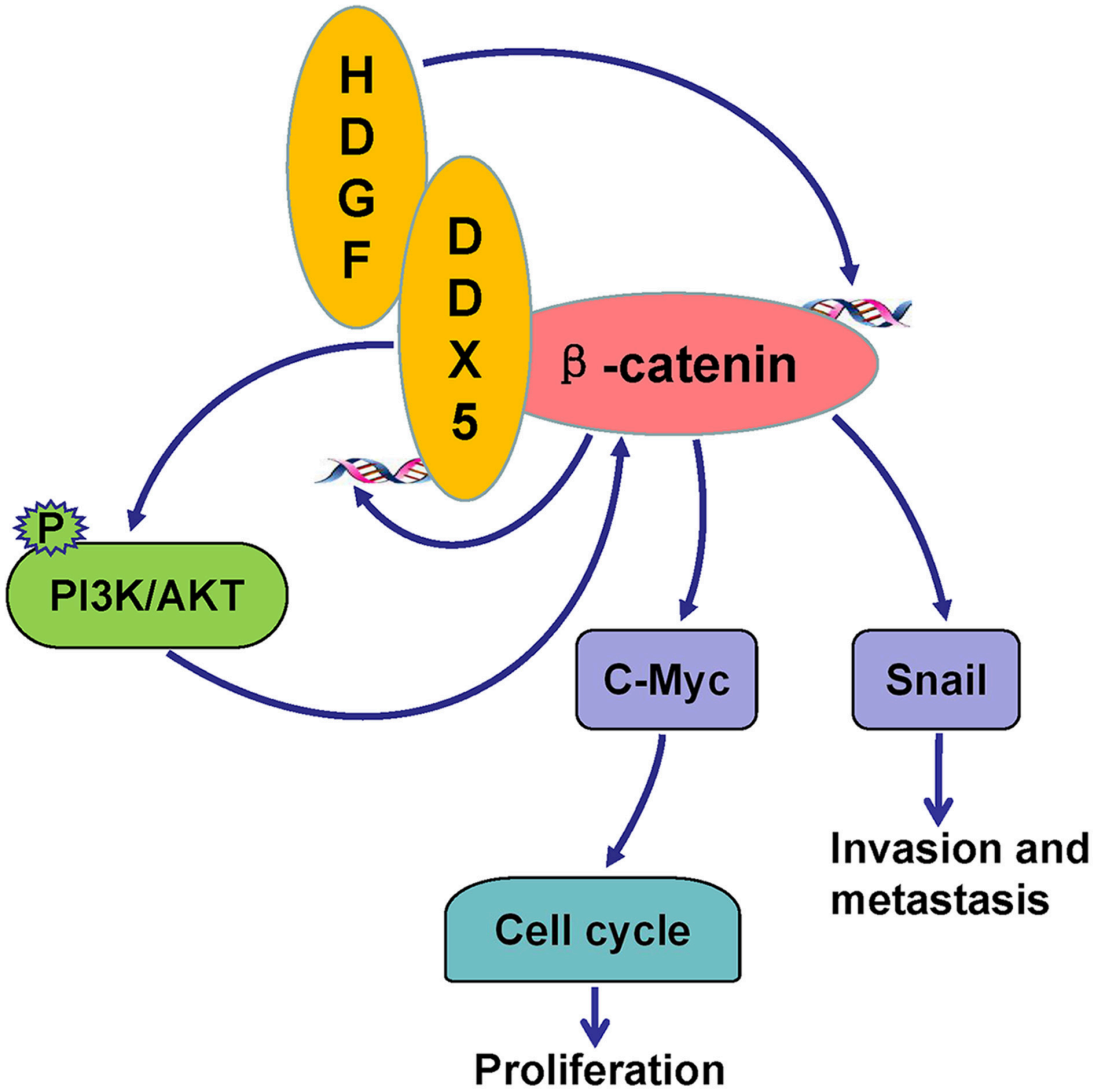
Potential signaling pathways utilized by HDGF/DDX5/β-catenin to promote proliferation, invasion, and metastasis in EC.

In summary, this study provides compelling evidence that HDGF in combination with DDX5 induces β-catenin to form a complex, which significantly promotes EC cell proliferation, migration, invasion, and metastasis *in vitro* and *in vivo*. The underlying mechanism likely involves the activation of PI3K/AKT signaling and downstream β-catenin-mediated cell cycle and EMT signaling proteins (Figure 8). Our results suggest that HDGF–DDX5 and β-catenin work together to play important roles in EC carcinogenesis and progression.

## Ethics Statement

For the use of the human tissue specimens for research purposes, prior consent from the patients and approval from the Ethics Committees of the Third Affiliated Hospital of Guangzhou Medical University were obtained. All animal studies were conducted in accordance with the principles and procedures outlined in the Southern Medical University Guide for the Care and Use of Animals.

## Author Contributions

CL, LW, QJ, and JZ conducted the research. SG and WF designed the research study. CL, LZ, LL, HJ, DL, and YX performed the statistical analysis. CL, LW, LZ, and WF wrote the manuscript. All authors have read and approved the final manuscript.

### Conflict of Interest Statement

The authors declare that the research was conducted in the absence of any commercial or financial relationships that could be construed as a potential conflict of interest.

## Abbreviations

co-IP: co-immunoprecipitation
DDX5: DEAD-box RNA helicase
EC: endometrial cancer
EMT: epithelial-mesenchymal transition
HDGF: hepatoma-derived growth factor
MTT, 3-(4 5-dimethylthiazol-2-yl)-2: 5-diphenyltetrazolium bromide
PBS: phosphate buffered saline
PCNA: proliferating cell nuclear antigen
PI3K: phosphoinositide 3-kinase
qRT-PCR: quantitative real-time reverse transcription polymerase chain reaction.
