# Supplementary material for: Hepatoma-Derived Growth Factor and DDX5 Promote Carcinogenesis and Progression of Endometrial Cancer by Activating β-Catenin
Source: Front Oncol. 2019 Apr 11;9:211. doi: 10.3389/fonc.2019.00211 (PMC6470266; doi:10.3389/fonc.2019.00211)
Supplement: Supplementary file 1 [file Table_1.docx]

Supplementary Table 1. shRNA sequences for HDGF.

| gene | No. |  | sequence |
| --- | --- | --- | --- |
| shHDGF | 1 | Sense | 5’ CcggtgCCGTGAAATCAACAGCCAACTCGAG TTGGCTGTTGATTTCACGGCATTTTTg 3’ |
|  |  | Antisense | 5’aattcaaaaatgCCGTGAAATCAACAGCCAACTCGAG TTGGCTGTTGATTTCACGGCA 3’ |
|  | 2 | Sense | 5’CcgggaACGAGAAAGGAGCGTTGAACTCGAGTTCAACGCTCCTTTCTCGTTCTTTTTg 3’ |
|  |  | Antisense | 5’aattcaaaaagaACGAGAAAGGAGCGTTGAACTCGAGTTCAACGCTCCTTTCTCGTTC 3’ |
|  | 3 | Sense | 5’CcggcgAGAACAACCCTACTGTCAACTCGAGTTGACAGTAGGGTTGTTCTCGTTTTTg 3’ |
|  |  | Antisense | 5’aattcaaaaacgAGAACAACCCTACTGTCAACTCGAGTTGACAGTAGGGTTGTTCTCG 3’ |
