# Supplementary material for: Hepatoma-Derived Growth Factor and DDX5 Promote Carcinogenesis and Progression of Endometrial Cancer by Activating β-Catenin
Source: Front Oncol. 2019 Apr 11;9:211. doi: 10.3389/fonc.2019.00211 (PMC6470266; doi:10.3389/fonc.2019.00211)
Supplement: Supplementary file 2 [file Table_2.docx]

Supplementary Table 2. Antibodies used for western blotting, immunohistochemical staining, immunofluorescence, and co-immunoprecipitation.

| Name of antibody | Cat.No | Company | Mol weight | Dilution (WB/IP/IF/IHC) |
| --- | --- | --- | --- | --- |
| HDGF | 60064-1-Ig | [Proteintech](http://www.so.com/link?url=http%3A%2F%2Fwww.ptglab.com%2F&q=PTG&ts=1460351828&t=0b49dce0d59f6db33e711e7d1a5a229&src=haosou) | 40 kDa | 1:1000 (WB); 1:100 (IF) ;1:100 (IHC) |
| HDGF | [11344-1-AP](http://www.ptgcn.com/Products/HDGF-Antibody-11344-1-AP.htm) | [Proteintech](http://www.so.com/link?url=http%3A%2F%2Fwww.ptglab.com%2F&q=PTG&ts=1460351828&t=0b49dce0d59f6db33e711e7d1a5a229&src=haosou) | 40 kDa | 1:200 (IP) |
| DDX5 | ab126730 | abcam | 69 kDa | 1:1000 (WB);1:100 (IP);1:200 (IF) ;1:100 (IHC) |
| β-catenin | 51067-2-AP | [Proteintech](http://www.so.com/link?url=http%3A%2F%2Fwww.ptglab.com%2F&q=PTG&ts=1460351828&t=0b49dce0d59f6db33e711e7d1a5a229&src=haosou) | 92 kDa | 1:1000 (WB);1:100 (IP);1:200 (IF) |
| P-Rb | 8180S | CST | 110kDa | 1:1000 (WB) |
| E2F1 | 3742S | CST | 70 kDa | 1:1000 (WB) |
| CCND1 | ab134175 | abcam | 36 kDa | 1:1000 (WB) |
| CDK4 | sc-260 | Santa | 34 kDa | 1:200 (WB) |
| c-Myc | ab32072 | abcam | 57 kDa | 1:1000 (WB) |
| P27 | 25614-1-AP | [Proteintech](http://www.so.com/link?url=http%3A%2F%2Fwww.ptglab.com%2F&q=PTG&ts=1460351828&t=0b49dce0d59f6db33e711e7d1a5a229&src=haosou) | 27 kDa | 1:1000 (WB) |
| PI3K | 60225 | PTG | 85 kDa | 1:2000-1:10000 (WB) |
| P-PI3K | 11508 | SAB | 55 and 85 kDa | 1:500-1:1000 (WB) |
| AKT | 60203 | PTG | 56-62 kDa | 1:2000-1:10000 (WB) |
| P-AKT | S473 | CST | 60 kDa | 1:2000 (WB) |
| E-cadherin | 20874-1-AP | [Proteintech](http://www.so.com/link?url=http%3A%2F%2Fwww.ptglab.com%2F&q=PTG&ts=1460351828&t=0b49dce0d59f6db33e711e7d1a5a229&src=haosou) | 125 kDa | 1:1000 (WB) |
| N-cadherin | 22018-1-AP | [Proteintech](http://www.so.com/link?url=http%3A%2F%2Fwww.ptglab.com%2F&q=PTG&ts=1460351828&t=0b49dce0d59f6db33e711e7d1a5a229&src=haosou) | 130 kDa | 1:1000 (WB) |
| vimentin | 3390S | CST | 57 kDa | 1:1000 (WB) |
| snail | 3879S | CST | 29 kDa | 1:1000 (WB) |
| PCNA | 10205-2-AP | [Proteintech](http://www.so.com/link?url=http%3A%2F%2Fwww.ptglab.com%2F&q=PTG&ts=1460351828&t=0b49dce0d59f6db33e711e7d1a5a229&src=haosou) | 36-38 kDa | 1:50 (IHC) |
| Ki67 | Ab16667 | abcam | ----- | 1:100 (IHC) |
| β-actin | sc-1616 | Santa | 43 kDa | 1:1000 (WB) |
| Flag-Tag | F1804 | Sigma-Aldrich | ----- | 1:100 (Co-IP) |
| His-Tag | 12698s | CST | ----- | 1:100 (Co-IP) |
