# Supplementary material for: Hepatoma-Derived Growth Factor and DDX5 Promote Carcinogenesis and Progression of Endometrial Cancer by Activating β-Catenin
Source: Front Oncol. 2019 Apr 11;9:211. doi: 10.3389/fonc.2019.00211 (PMC6470266; doi:10.3389/fonc.2019.00211)

Supplementary Figure 1. Ishikawa and RL95-2 cells display green fluorescence after infection with a virus expressing HDGF.


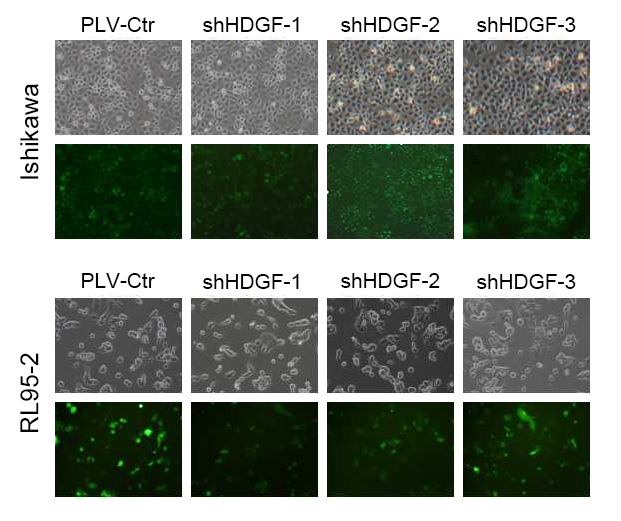


Supplementary Figure 2: Similar HDGF interference results.


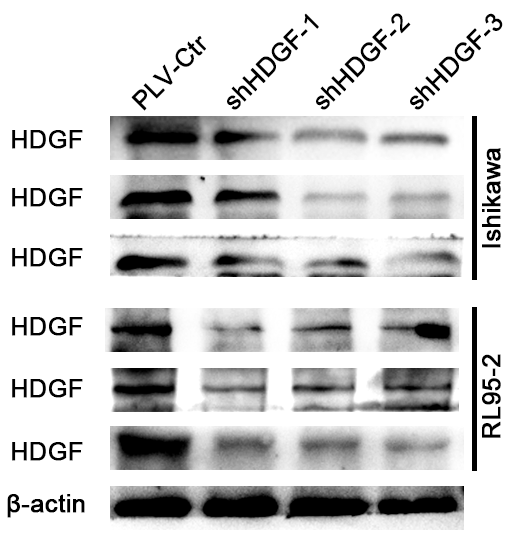

Supplement: Supplementary file 4 [file Data_Sheet_1.docx]
